# Organic amendments with high proportion of heterocyclic compounds promote soil microbiome shift and microbial use efficiency of straw-C

**DOI:** 10.3389/fmicb.2023.1087709

**Published:** 2023-01-18

**Authors:** Jingwang Li, Lin Chen, Jiabao Zhang, Congzhi Zhang, Donghao Ma, Guixiang Zhou, Qi Ning

**Affiliations:** ^1^Fengqiu Experimental Station of National Ecosystem Research Network of China, State Key Laboratory of Soil and Sustainable Agriculture, Institute of Soil Science, Chinese Academy of Sciences, Nanjing, China; ^2^University of Chinese Academy of Sciences, Beijing, China

**Keywords:** organic amendments, heterocyclic compounds, microbial community, microbial use efficiency of straw-C, pyrolysis-GC/MS

## Abstract

Soil microbial use efficiency of straw carbon (C), which is the proportion of straw-C microbes assimilate into new biosynthetic material relative to C lost out of the system as CO_2_, is critical in increasing soil organic C (SOC) content, and hence maintaining soil fertility and productivity. However, the effect of chemical structures of the organic amendments (OAs) on the microbial use efficiency of straw-C remains unclear. The effect of the chemical structure of the OAs on microbial use efficiency of straw-C was elucidated by a combination of ^13^C-straw labeling with high-throughput sequencing and pyrolysis-GC/MS. We found a strong positive correlation between the microbial use efficiency of straw-C and the proportion of heterocyclic compounds (Hete_C). The microbial use efficiency of straw-C was highest in soil supplemented with Hete_C-dominant OAs, which significantly shifted microbial community structure toward fungal dominance. Specifically, fungal-to-bacterial ratio, fungal richness, and the relative abundance of *Ascomycota* were higher in soil with a higher proportion of Hete_C-dominant OAs. Together, our study suggests that OAs with high proportion of Hete_C promote the microbial use efficiency of straw-C by increasing the dominance of fungi in the soil microbial community in agroecosystems.

## Introduction

Soil organic carbon (SOC) is crucial in maintaining soil fertility and productivity, and straw returning is an important strategy to improve SOC in agroecosystems (Lal, 2004). Therefore, it is critical to enhancing the efficiency of straw carbon (C) conversion into SOC. Soil microorganisms convert straw-C to SOC and mineralized carbon dioxide (CO_2_; Cebrian and Lartigue, 2004). During the decomposition of straw, microorganisms use part of the assimilated C for growth, which significantly contributes to SOC, and use part of the assimilated C for CO_2_ mineralization during respiration (Liang et al., 2017). The microbial use efficiency of straw-C is the growth ratio to the amount of assimilated C. Therefore, the microbial use efficiency of straw-C is essential in improving SOC in agricultural systems.

In general, microbial C use efficiency (CUE) depends on the soil microbial community structure (Sinsabaugh et al., 2013; Kaiser et al., 2014). Bacteria and fungi are the main decomposers of organic matter in the soil, but both have different life strategies and metabolisms (Strickland and Rousk, 2010). Previous studies reveal that fungi make greater contribution to SOC accumulation than bacteria (Six et al., 2006; Strickland and Rousk, 2010). Presumably, microbial CUE is higher in microbial communities with higher fungal-to-bacterial ratios (F:B). However, recent investigations have demonstrated that microbial CUE is higher in a microbial community with lower F:B (Silva-Sánchez et al., 2019; Soares and Rousk, 2019). Thus, whether the microbial use efficiency of straw-C is higher in a fungi-dominated community or in a bacterial-dominated community in agricultural soils remains controversial. Additionally, microbial CUE is affected by the relationships among microbial communities (Geyer et al., 2016). Biological relationships between fungi and bacteria alter community-level functions by affecting the community composition and directly or indirectly altering the behavior and physiology of certain microbial species (Mitri and Foster, 2013). The effects of the complex community relationships on the microbial use efficiency of straw-C remain elusive.

The current main approach to improving the microbial CUE in agricultural soils relies on the shift of the microbial community structure by adding organic amendments (OAs; Liu et al., 2020). The quality of OAs added to the soil affects the structure of the microbial community, which indirectly affects the microbial CUE (Zhong, 2020). The OAs for soils comprise varying proportions of recalcitrant compounds and labile compounds (KoÈgel-Knabner, 2002). Different organic compounds favor the colonization of specific microbial communities (Torres et al., 2014). Some studies have shown that the microorganisms favored by OAs rich in recalcitrant compounds have a high microbial CUE (Liu et al., 2020). In contrast, other studies show that the specific microbial community recruited by OAs with a high proportion of labile compounds has a high microbial CUE (Miao et al., 2021). Thus, the relationships between the quality of OAs and the microbial CUE (i.e., microbial use efficiency of straw-C) in agricultural soils remain controversial.

Herein, we investigated the effect of the type of OAs on the soil microbial community and microbial use efficiency of straw-C. The chemical structure of the OAs was analyzed using pyrolysis gas-chromatography/mass-spectrometry (pyrolysis-GC/MS). Pyrolysis-GC/MS is a powerful technology for exploring the molecular composition of complex OAs (Yang et al., 2007; Gonzalez-Vila et al., 2009; Kaal et al., 2016). The microbial use efficiency of straw-C was calculated by ^13^C-straw labeling. The bacterial and fungal community structures were elucidated by amplicon sequencing of the 16S rRNA and internal transcript spacer (ITS) genes, respectively. In addition, the abundance of bacterial and fungal cellulose-degrading genes was quantified. We hypothesized that OAs with a higher proportion of labile compounds cause a significant shift in the soil microbial community structure toward fungal dominance, enhancing the microbial use efficiency of straw-C.

## Materials and methods

### Soil and organic amendments

Soil was sampled from the Yangliu Agro-Ecological Experiment Station, Suixi County, Anhui Province, China (33°37′ N, 116°45′ E) in June 2020 (immediately following the harvest of winter wheat). The samples were collected from the top 20-cm soil layer using an auger, air-dried, and passed through a 2-mm sieve. The soil was classified as a Shajiang Calci-Aquic Vertosol according to the Chinese Soil Taxonomy. The soil contained 14.24 g kg^−1^ of organic C, 0.99 g kg^−1^ of total N, and 0.67 g kg^−1^ of total P (Li et al., 2014).

We selected three types of OAs which comprise lignin-dominant OAs (LM), cellulose-dominant OAs (CM), and OAs with equal content of lignin and cellulose (MM). The molecular structure of OAs was determined using pyrolysis-GC/MS (MDGCMSMS TQ8050, SHIMADZU, Japan). All identifiable pyro-lysates were categorized into eight chemical groups: lignin compounds, phenol compounds, carbohydrates, saturated hydrocarbons, unsaturated hydrocarbons, nitrogen-containing compounds, aromatic hydrocarbons, and poly-aromatic hydrocarbons, according to the degree of chemical similarity; and four types of organic compounds were further classified based on chemical structure of organic C skeletons: alicyclic compounds (Alic_C), aliphatic compounds (Alip_C), aromatic compounds (Arom_C), and heterocyclic compounds (Hete_C; Supplementary Table S1).

### Soil incubation experiment

Soil was added with LM, MM, or CM, and soil without addition of OAs was used as the control (Ctrl). The experiment included four treatments with each six replicates. OA was added to 80 g of soil (oven-dry weight) at a rate of 5 mg C g^−1^ soil (Savy et al., 2020), and then pre-incubated at 25°C in dark for 30 days. N and P nutrients were provided according to C:N:P stoichiometric ratios (C:N:*p* = 10,000:833:200; Kirkby et al., 2013). Soil moisture was adjusted to 60% of water-holding capacity (WHC) using sterile deionized water during the incubation period. After the pre-incubation, the three replicates of each treatment received ^13^C-labeled straw (δ^13^C 99‰), and another three replicates without addition of ^13^C-straw were used to partition the mineralization and microbial assimilation of the ^13^C-straw. The addition rate was 1 mg straw-C g^−1^ soil, equivalent to the amount of straw returned into soils per year. An open scintillation vial containing 10 ml of 3 M sodium hydroxide (NaOH) was placed to absorb CO_2_ respired during the incubation. All soils were incubated at 25°C and 60% WHC for 28 days. Previous studies have demonstrated that soil microbes efficiently used easily-degradable component of straw in 1 week and labile straw-C was almost anabolized by soil microbes in a month (Chen et al., 2014; Zhong, 2020). Therefore, the soils were sampled in 7 and 28 days of the incubation to analyze CO_2_, microbial biomass C (MBC), 16S rRNA and ITS genes, and the abundance of cellulose-degrading genes.

### Soil chemical analysis

CO_2_ was trapped in 10 ml 3 M NaOH trap solution to measure soil respiration at 7 and 28 days of incubation as previously described (Zibilske, 1994). BaCO_3_ pellets were dried at 50°C and stored for ^13^C analysis using an isotope ratio mass spectrometer (Delta Plus, IRMS; Thermo Fisher Scientific, Bremen, Germany). MBC at 7 and 28 days of incubation was determined using the chloroform fumigation-extraction method (Makarov et al., 2015). For ^13^C measurement of microbial biomass, an aliquot (*ca.* 10 mL) of the K_2_SO_4_ extract was freeze-dried, and the solid material was then analyzed.

### Determining microbial use efficiency of straw-C

The microbial use efficiency of straw-C is the ratio of C incorporated in microbial biomass and used for respiration. The C used for respiration was based on the accumulation of CO_2_ in 7 or 28 days. The proportion (f%) of CO_2_-C or MBC derived from ^13^C-straw was calculated using a two-component isotopic mixing model based on Eq. (1) (Balejdent and Balabane, 1992):


(1) $$f\left(\%\right)=\frac{\delta -\delta_{soil}}{\delta_{straw}-\delta_{soil}}$$

where δ is the δ^13^C of CO_2_-C or MBC in the ^13^C-straw amended soils (‰), δ_soil_ is the δ^13^C of the ^13^C-straw treatments (‰), and δ_straw_ is the δ^13^C of the ^13^C-straw (‰).

The δ ^13^C of MBC was calculated using Eq. (2) (De et al., 2011):


(2) $$\delta^{13}C_{MBC}=\frac{\delta C_{fum}\times C_{fum}-\delta C_{nfum}\times C_{nfum}}{C_{fum}-C_{nfum}}$$

where δC_fum_ and δC_nfum_ are the δ^13^C levels in fumigated and non-fumigated soil extracts, respectively, and C_fum_ and C_nfum_ are the total MBC contents of the fumigated and non-fumigated soil extracts, respectively.

The CO_2_ emission or MBC amount in the ^13^C-straw was calculated by multiplying f% with the total CO_2_ emission or total MBC amount.

The microbial use efficiency of straw-C was calculated according to Eq. (3) (Geyer et al., 2016):


(3) $$\;\mathrm{Microbial}\;\mathrm{use}\;\mathrm{efficiency of straw}-C=\frac{C13-MBC}{C13-MBC+C13-CO_{2}}$$

where ^13^C-MBC is the MBC derived from ^13^C-straw (mg kg^−1^ soil), and ^13^C-CO_2_ is the cumulative ^13^C-straw (mg kg^−1^ soil) mineralized by day 7 or 28.

### DNA extraction, amplicon sequencing of 16S rRNA and ITS genes, and determination of the abundance of cellulose-degrading genes

DNA was extracted from 0.25 g fresh soil samples taken at days 7 and 28. The extraction was done using the Power Soil DNA isolation kit (MoBio, Carlsbad, CA, United States), according to the manufacturer’s instructions. The DNA concentrations were determined by a nanodrop spectrophotometer (NanoDrop, Wilmington, DE). The Illumina sequencing was done by Novogene. The fragments of bacterial 16S rRNA gene and fungal internal transcribed spacer (ITS) gene were amplified using the primer pairs 341F (5′-CCTAYGGGRBGCASCAG-3′)/806R (5′-GGACTACNNGGGTATCTAAT-3′) and ITS1F (5′-CTTGGTCATTTAGAGGAAGTAA-3′)/ITS2R (5′-GCTGCGTTCTTCATCGATGC-3′), respectively. A sample-specific barcode was added to the forward primer for distinguishing the amplicons from different samples. The PCR conditions were 94°C for 5 min; 30 cycles of 94°C for 30 s, 52°C for 30 s, and an extension of 72°C for 30 s, followed by 72°C for 10 min, and held at 4°C. PCR products from all samples were pooled and purified in equimolar concentrations, and sequencing was performed on an Illumina MiSeq instrument (Caporaso et al., 2012). The bacterial 16S rRNA gene sequencing and ITS gene sequencing data were uploaded to the NCBI SRA database under accession number PRJNA898503.^1^

The raw FASTQ files were demultiplexed and quality-filtered using QIIME2. Subsequently, the amplicon variant sequences (ASVs) with a 97% similarity were clustered together using DADA2 (version 1.8). Poor-quality sequences of less than 200 bp and quality scores of less than 20 were removed, and the chimeras were removed using the UCHIME algorithm (Edgar et al., 2011). The phylogenetic affiliation of each rRNA gene sequence was determined using the UNITE database for the ITS1 rRNA region and the Ribosomal Database Project (RDP) Classifier^2^ against the SILVA (SSU117/119) database for the 16S rRNA gene at an 80% confidence threshold (Wang et al., 2007).

The fungal *cbhI* gene and bacterial *GH48* gene were selected as functional biomarkers of cellulolytic fungi and bacteria, respectively. The primers for quantitative PCR (qPCR) of bacterial GH48 and fungal cbhI genes have been described in a previous study (Edwards et al., 2008). The primers *GH48 F8/GH48 R5* (*GH48_F8*: 5 GCCADGHTBGGCG ACTACCT 3; *GH48_R5*: 5 CGCCCCABGMSWWGTACCA 3) and *cbhI F*/*cbhI R* (*cbhI F*: ACCAAYTGCTAYACIRGYAA; *cbhI R*: GCYTCCCAIATRTCCATC) were used for the qPCR assay. The abundance of bacterial *GH48* and fungal *cbhI* genes was quantified according to modified procedures (Zhang et al., 2017). The thermal profiles of qPCR for the target genes of *GH48* and *cbhI* were as follows: 95°C for 5 min, 40 × (94°C for 30 s, 60°C for 45 s, and 72°C for 90 s), and data collection at 84°C for 10 s; and 94°C for 4 min, 40 × (94°C for 45 s, 50°C for 30 s, and 72°C for 60 s), and data collection at 81°C for 10 s, respectively. The initial concentrations of the two plasmids as the standards for bacterial *GH48* and fungal *cbhI* gene abundance analysis corresponded to 1.85 × 10^11^ and 2.65 × 10^10^ copies g^−1^ dry soil, respectively.

### Statistical analysis

The effect of different OAs on microbial use efficiency of straw-C, fungal-to-bacterial (F:B) ratio and *cbhI-to-GH48* ratios was analyzed using a one-way analysis of variance (ANOVA). Statistical significance was set at *p* < 0.05. Principal coordinate analysis (PCoA) was performed based on Bray-Curtis distances to visualize the change in the bacterial and fungal community under different OAs (Bray and Curtis, 1957). The difference in microbial use efficiency of straw-C among the OAs groups was analyzed using ANOVA. A correlation matrix was constructed to describe the complex co-occurrence patterns in bacteria-fungi networks. The maps were created using the geom_polygon function in the “ggplot2” package in R software.

Co-occurrence networks of bacteria and fungi in different OAs groups were constructed (Faust and Raes, 2012). We focused on the most dominant phylotypes, both abundant (with relative abundance higher than 0.1%) and ubiquitous (bacteria and fungi were present above half of all the samples). The filtering identified dominant phylotypes in soils and minimized potential spurious correlations from the rare taxa. The focus was on the dominant soil phylotypes because they are expected to have disproportionate functional importance in their ecosystems and are widely distributed. A valid co-occurrence was considered a statistically robust correlation between species when the correlation coefficient (r) was >0.6 or < −0.6 at *p* < 0.01. The *p*-values < 0.01 were adjusted by the Benjamini-Hochberg procedure to reduce the chances of obtaining false positives. The network modules were identified using the “igraph” package. The number and relative abundance of ASVs in the three most important modules were calculated to assess the effects of different OAs on specific phylotypes with strong interconnections. In addition, subnetworks were extracted by preserving the phylotypes of individual soil samples using the subgraph function in “igraph” package. Networks were constructed and visualized using the “igraph” package. Random forest modelling was performed using the “random-forest” package, and the model significance and predictor importance were determined using the “rfPermute” packages. The rest of the analyses were performed with the R software.

## Results

### Effects of different OAs on microbial use efficiency of straw-C

**Figure 1:**
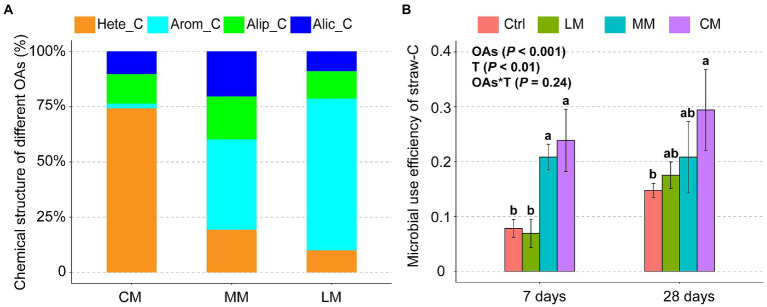
Effects of organic amendments (OAs) with different chemical structures on microbial use efficiency of straw-C. **(A)** The chemical structure of different OAs. **(B)** The microbial use efficiency of straw-C under different OAs. Data are presented as means ± SD (*n* = 3). Error bars depict standard deviations. Lowercase letters indicate significant differences (*p* < 0.05) between the treatments. Ctrl, Control without OAs; LM, Addition of lignin-dominant OAs; MM, Addition of OAs with equal content of lignin and cellulose; and CM, Addition of cellulose-dominant OAs.

Py-GC/MS analysis revealed that the three OAs had considerably different chemical structures (Figure 1A). Hete_C was predominant in the CM, Arom_C was predominant in the LM, and Hete_C and Arom C were in equal proportions in the MM. The microbial use efficiency of straw-C was significantly (*p* < 0.001) higher in Hete_C rich CM compared with other treatments in 7 days. There was no significant difference in microbial use efficiency of straw-C among the three OAs treatments in 28 days. Therefore, straw-C decomposition efficiency was higher in the CM than in the LM and MM (Figure 1B).

### Effects of different OAs on bacterial and fungal community structures

**Figure 2:**
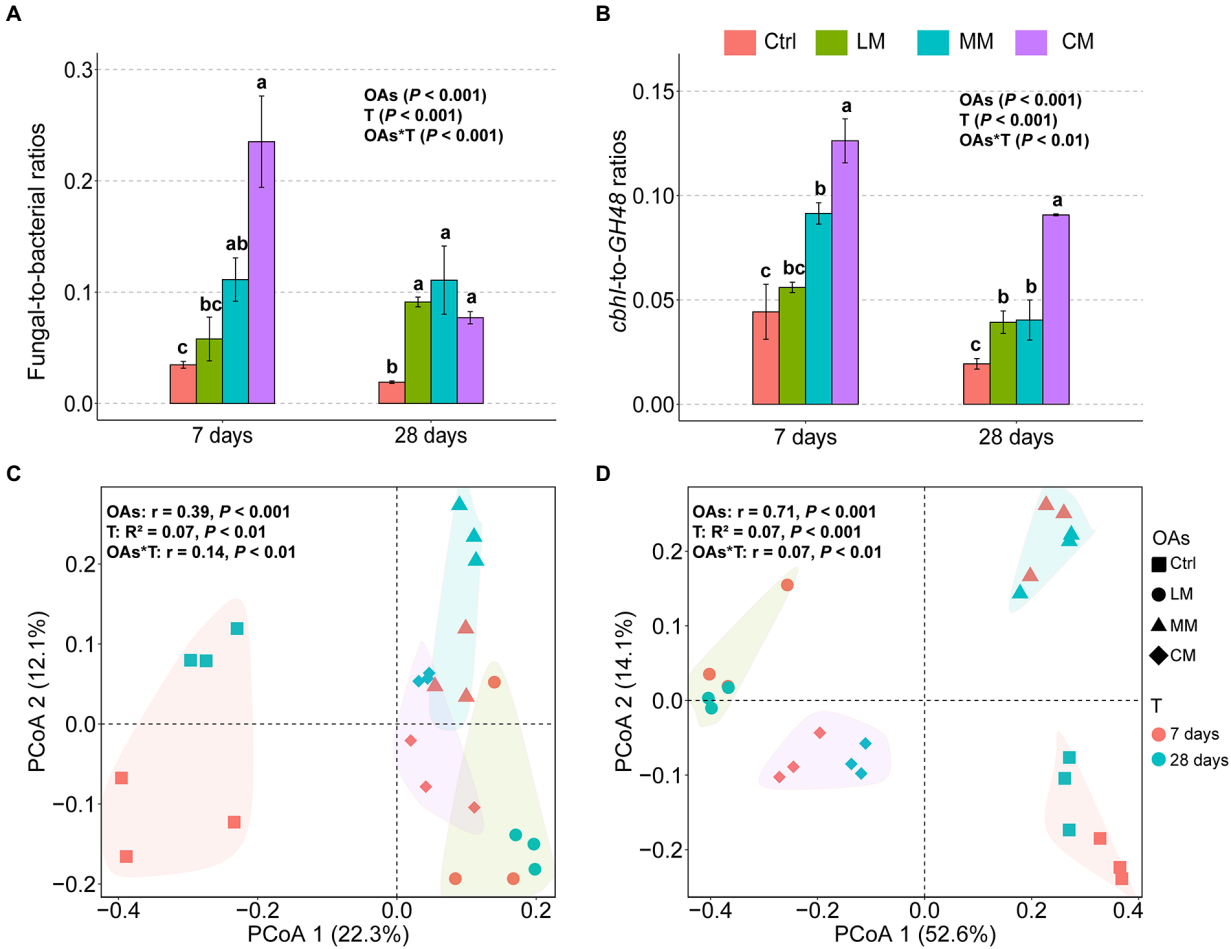
Effects of organic amendments (OAs) with different chemical structures on fungal-to-bacterial ratios (F:B) and their *C-cycling* gene ratios (*cbhI-to-GH48* ratios) as well as bacterial and fungal diversity. **(A)** The fungal-to-bacterial ratios (F:B) under different OAs. **(B)** The *cbhI-to-GH48* ratios (*cbhI:GH48*) under different OAs. Data are expressed as means ± SD (*n* = 3). Error bars depict standard deviations. Lowercase letters indicate significant differences (*p* < 0.05) between the treatments. Principal coordinates analysis (PCoA) of the bacterial **(C)** and fungal **(D)** communities with different OAs supplements at days 7 and 28 based on Bray-Curtis distance (d). The polygons indicate a 95% standard error for each treatment. Ctrl, Control without OAs; LM, Addition of lignin-dominant OAs; MM, Addition of OAs with equal content of lignin and cellulose; and CM, Addition of cellulose-dominant OAs.

The chemical structures of OAs significantly influenced the microbial community structure (Figure 2). The F:B ratios and *cbhI-GH48* were higher in the CM (*p* < 0.001), which all declined over time (Figures 2A,B). F:B ratio declined because the abundance of fungi decreased sharply over time, whereas the abundance of bacteria increased (Supplementary Table S2). The *cbhI-GH48* ratios declined because even though the abundance of both *cbhI* and *GH48* increased over time, the increase in expression of *GH48* was higher than that of *cbhI* (Supplementary Table S2).

There was no significant difference in the bacterial richness among the three OAs (Supplementary Figure S1A, *p* = 0.09), but fungi richness was considerably lower in the CM (Supplementary Figure S1B, *p* < 0.001). Principal coordinates analysis (PCoA) revealed that both fungi and bacteria diversity differed considerably among the three types of OAs (*p* < 0.001; Figures 2C,D). Furthermore, the degree of fungal partitioning (R^2^ = 0.71) was greater than bacterial partitioning (R^2^ = 0.39). In addition, the type of OAs had little effect on the relative abundance of bacteria at the phylum level (Supplementary Figure S2A) but significantly impacted the abundance of fungi at the phylum level (Supplementary Figure S2B). Specifically, OAs with high Hete_C proportion favored a significant increase in the relative abundance of *Ascomycota*.

### Effects of different OAs on bacterial and fungal co-occurrence patterns

**Figure 3:**
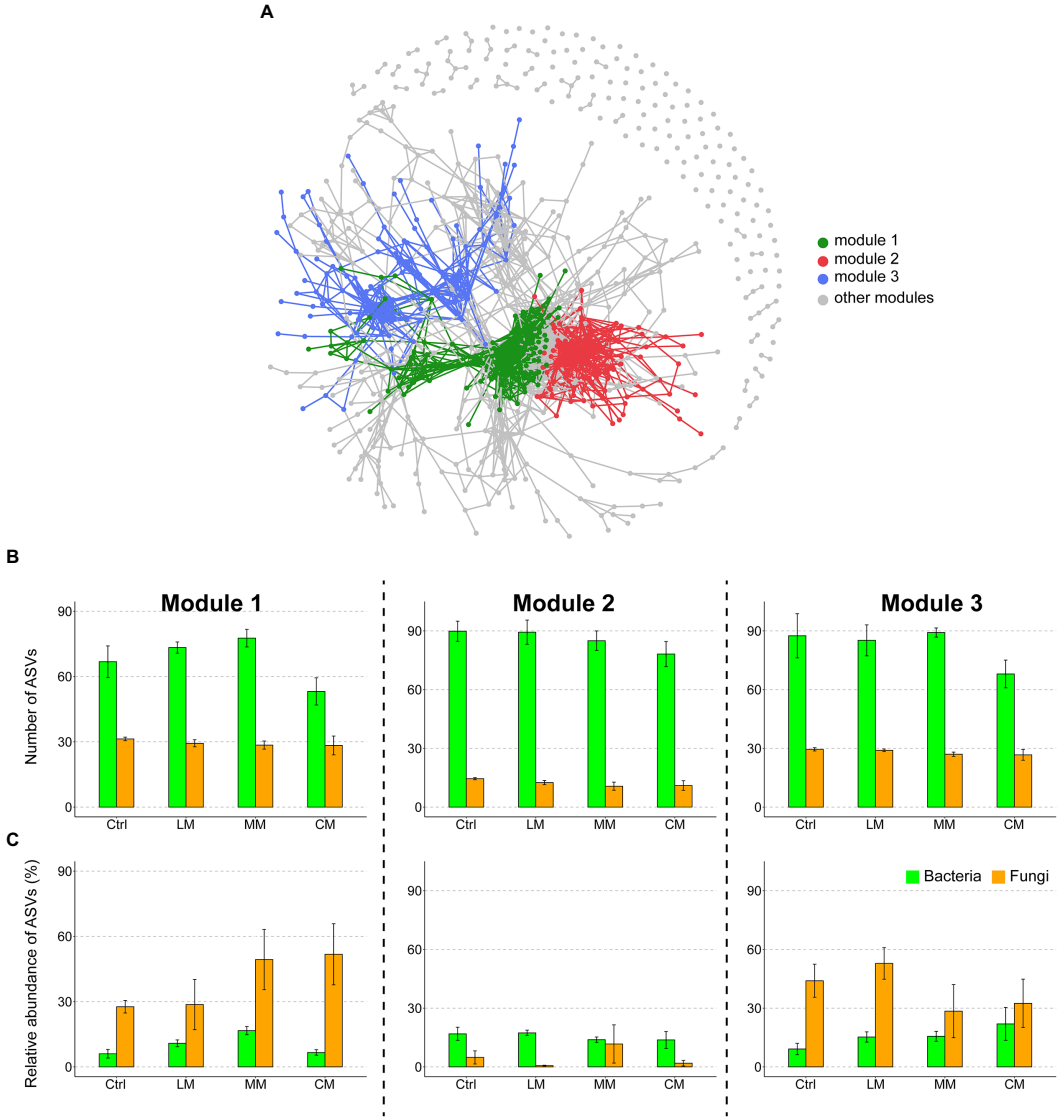
**(A)** Network co-occurrence patterns of the dominant bacteria and fungi across treatments. The modules are distinguished using different colors. **(B)** The number of amplicon variant sequence (ASVs) of bacteria and fungi in the three most important modules (modules 1–3). **(C)** Relative abundance of ASVs of bacteria and fungi in the three most important modules (modules 1–3) in different OAs treatments. Data are presented as means ± SD (*n* = 6). Error bars depict standard deviations. Lowercase letters indicate significant differences (*p* < 0.05) between the treatments. Ctrl (Control without OAs), LM (Addition of lignin-dominant OAs), MM (Addition of OAs with equal content of lignin and cellulose), and CM (Addition of cellulose-dominant OAs).

To evaluate the effects of different OAs on the microbial co-occurrence patterns, we built a correlation-based network incorporating the dominant bacterial and fungal phylotypes. We identified three highly connected modules within the network (Figure 3A). The number of bacteria was larger than that of fungi in the three most important modules (i.e., modules 1–3; Figure 3B). The relative abundance of fungi was higher than that of bacteria in modules 1 and 3, while an opposite trend existed in module 2 (Figure 3C). Compared with the Ctrl and LM, the CM drastically increased the relative abundance of fungi in module.

### The relationship between organic compounds in the OAs and bacterial and fungal community structures and microbial use efficiency of straw-C

**Figure 4:**
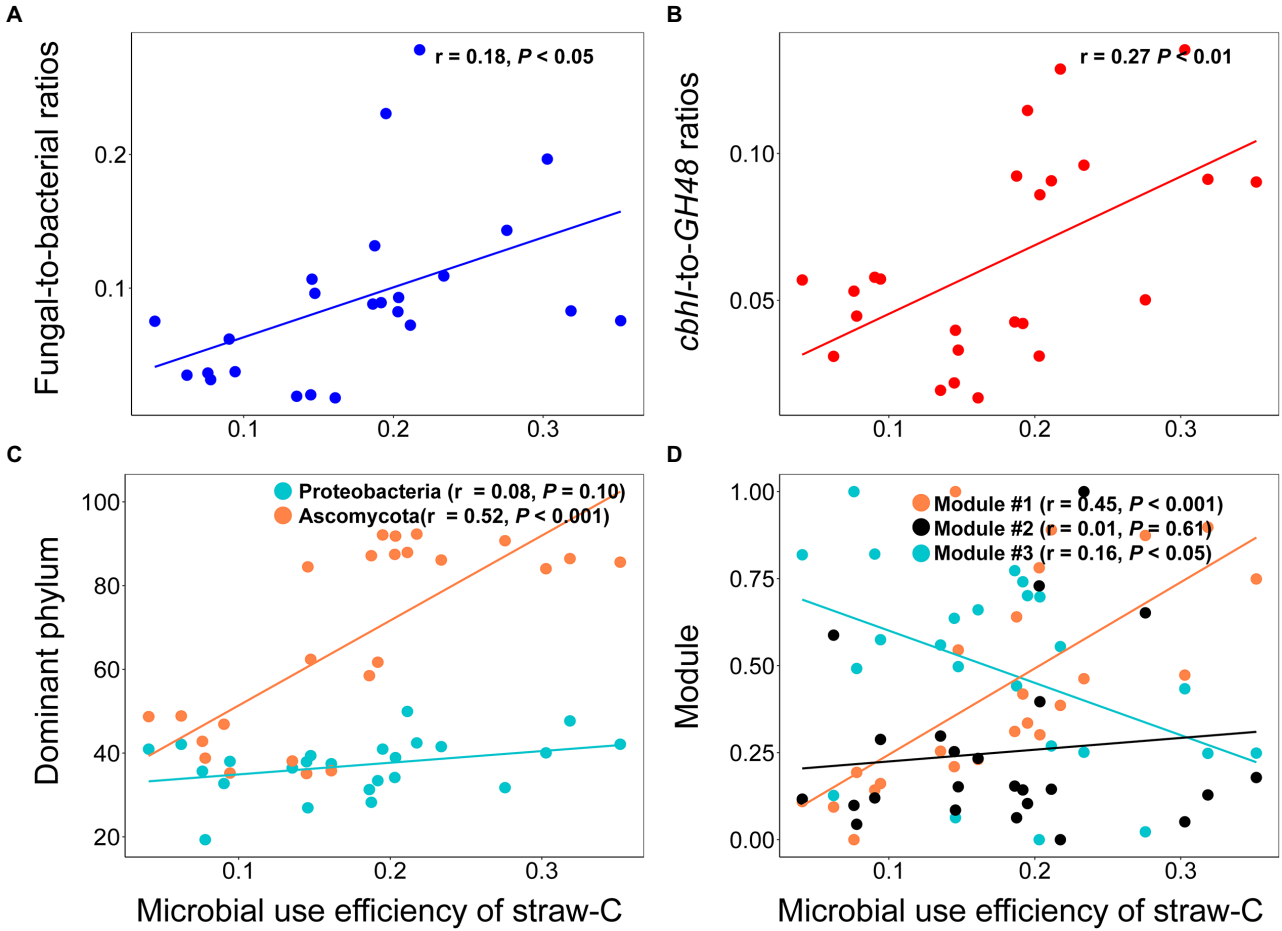
Effects of fungal-to-ratios **(A)**, *cbhI-to-GH48* ratios **(B)**, dominant phylum **(C)**, and three main ecological clusters **(D)** on microbial use efficiency of straw-C.

**Figure 5:**
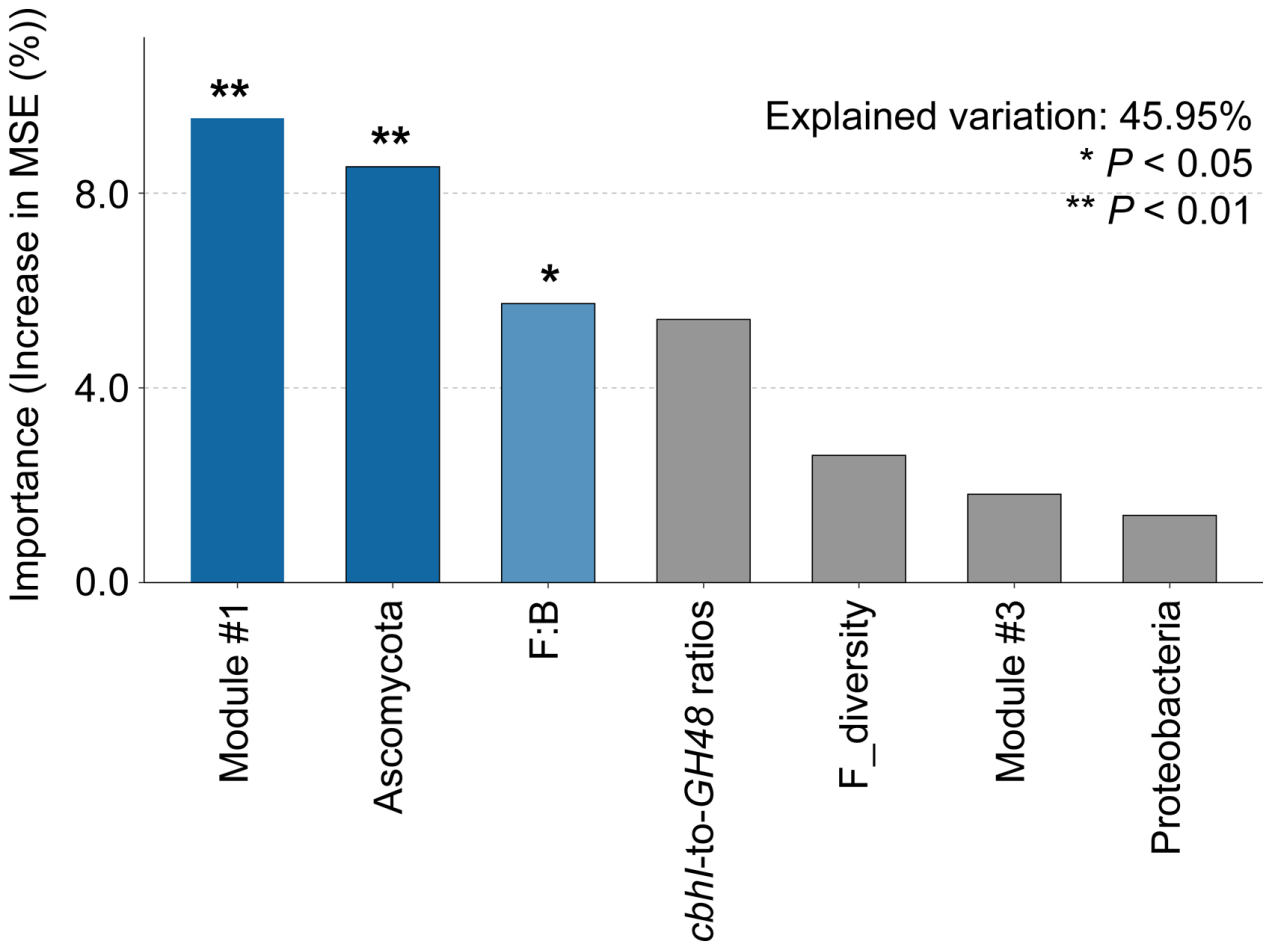
Mean predictor importance of the relative abundances of indicators of microbial community properties on the microbial use efficiency of straw-C. The contribution (% of increased mean square error) is calculated based on random forest analyses. Percentage increases in the MSE (mean squared error) of variables estimate the importance of these predictors. The MSE% values were directly proportional to the importance of a given predictor. *p < 0.05, **p < 0.01.

The type of OAs influenced the microbial community indices and microbial use efficiency of straw-C. Hete-C and Arom_C reduced the microbial community indices (Supplementary Figure S3). Specifically, F:B, *cbhI-GH48*, *Ascomycota*, *Proteobacteria,* and microbial use efficiency of straw-C were strongly (*p* < 0.05) and positively correlated with Hete_C content but were strongly (*p* < 0.05) and negatively correlated with Arom_C content. Furthermore, in investigating the correlation of microbial community structure with microbial use efficiency of straw-C, we found that microbial use efficiency of straw-C increased significantly (*p* < 0.05) with F:B, *cbhI:GH48*, the abundance of module #1, and the relative abundance of *Ascomycota*, whereas there was a strong negative correlation between the abundance of module #3 members (*p* < 0.05) and microbial use efficiency of straw-C (Figure 4). In addition, there was a weak negative correlation between microbial use efficiency of straw-C and the abundance of *Proteobacteria,* but there was a strong negative correlation between the bacterial diversity (*p* < 0.05) and the fungal diversity (Supplementary Figure S4). The microbial community with greater microbial use efficiency of straw-C were predicted using random forest modelling. The importance of a variable was determined by removing it from the model and determining the subsequent change in the model’s mean square error (MSE). Our results indicated that module #1 was potentially more important in the microbial use efficiency of straw-C, accounting for 11.92% of the MSE (Figure 5, *p* < 0.01). Moreover, *Ascomycota* and F:B were the next most important factors influencing microbial use efficiency of straw-C, accounting for 10.68% (*p* < 0.01) and 7.16% (*p* < 0.05) respectively, of the MSE.

## Discussion

The addition of OAs with a high proportion of Hete_C significantly increased the microbial use efficiency of straw-C in the soil by shifting the microbial community. Similarly, Soares and Rousk (2019) reported that microbial CUE was directly proportional to the C-quality (i.e., plant litter with a high proportion of labile compounds). Likewise, Silva-Sánchez et al. (2019) also found that the growth rates of both bacteria and fungi, which indicate the ability of these organisms to assimilate C into their biomass, are stimulated by high-quality OAs. This might be due to the unique chemical structure of Hete_C, in which N or O atoms are embedded in the C rings (Anslyn and Dougherty, 2006; Ouellette and Rawn, 2018). This lowers chemical bonding energies relative to Arom_C compounds, making them more unstable and easily utilized by microorganisms. As a result, microorganisms use less energy to break down Hete_C into simple forms that can be utilized for growth and development. Therefore, soil microorganisms produced more cell mass per unit mass of straw-C broken down (i.e., soil microbial use efficiency of straw-C) in soil containing a high proportion of Hete_C-dominant OAs.

OAs influenced the microbial use efficiency of straw-C by altering microbial community structure. OAs used in the present research were complex natural macromolecular organic compounds. Thus, fungi, which degrade complex macromolecular compounds more efficiently than bacteria, were recruited (van der Wal et al., 2007; Challacombe et al., 2019; Clocchiatti et al., 2020). In the present study, we hypothesized that fungal groups would dominate soils with a high proportion of labile compounds (i.e., Hete_C) and OAs. The soil F:B and microbial use efficiency of straw-C were higher in soils with Hete_C-dominant OAs than other OAs. In contrast with Soares and Rousk (2019), we observed that microbial use efficiency of straw-C was strongly and positively correlated with Hete_C content and fungal abundances. This confirmed our hypothesis that the microbial use efficiency of straw-C is high in soils with higher fungal-to-bacterial ratios. Our results are consistent with Kallenbach et al. (2016) findings, who observed that microbial-driven SOM accumulation was very high in soils with higher fungal abundances. Similarly, Liu et al. (2020) found that microbial CUE was significantly high in soils abundant with fungi. In general, fungi are considered to metabolize organic compounds more effectively than bacteria (Malik et al., 2016) and thus have a higher CUE than bacteria. Furthermore, fungal cells are larger than bacterial cells and thus have a lower surface area to volume ratio (Six et al., 2006). Therefore, fungal necromass form macromolecular aggregations more efficiently, which decreases the availability of fungal necromass to be utilized by microbiome. However, for small molecular organic compounds like glucose which microbes can easily assimilate, the microbial CUE is still high with lower F:B (Eilers et al., 2010; Fu et al., 2022). This might be because bacteria are 10 to 100 times more abundant than fungi and mainly utilize small molecular substrates. We found that the F:B ratio decreased over time since the expression of bacterial genes increased more rapidly over time than fungal genes. The higher *cbhI-GH48* ratio during CM and the addition of OAs higher in Hete_C content corroborated the above results. In fungi, cellobiohydrolase is encoded by the *cbhI* gene (Edwards et al., 2008), while in bacteria, it is encoded by the *GH48* gene (Sizova et al., 2011). Cellobiohydrolase participates in cellulose degradation. Our findings showed that the *cbhI-GH48* ratios decreased over time because the bacterial abundance increases more rapidly than the fungal abundance. Further analyses confirmed that the F:B ratio decreased over time degradation. Therefore, F:B and microbial use efficiency of straw-C at the beginning of degradation was higher in soils with Hete_C, which is more complex than Arom C, but F:B decreased while microbial use efficiency of straw-C gradually increased with the decomposition of complex OAs.

Notably, the relative abundance of *Ascomycota* (the dominant fungi in CM and MM) increased in soils with high Hete_C content. The dominant fungi identified in CM and MM are widely distributed in soils worldwide, and their abundance is strongly and positively correlated with the microbial CUE (Ma et al., 2013; Challacombe et al., 2019). This suggests that *Ascomycota* plays a critical role in decomposing OAs and generating SOC, and might be because *Ascomycota* secretes numerous enzymes that degrade macromolecules (Lumbsch and Huhndorf, 2010). In addition, fungal richness decreased significantly reduced in OAs with a high proportion of Hete_C. However, members of *Ascomycota* increased in soils with OAs and a high proportion of Hete_C.

Additionally, we further found that a specific microbial community recruited by OAs contributes more to the microbial use efficiency of straw-C than single microorganism taxa by random forest modelling. This is consistent with previous studies which reported that microbes collaborate in breaking down and utilizing organic materials by exuding substances that stimulate the growth and reproduction of species that are favorable to their existence and dominance (Faust et al., 2017; Ning et al., 2020; Yuan et al., 2021). Fungi can degrade complex organic macromolecules into numerous small molecules using secretory extracellular enzymes such as cellulase and laccase (Clocchiatti et al., 2020). Except for the stronger ability of fungi to secrete extracellular enzymes, the large amounts of organic acids secreted by fungi also degrade straw (Wang et al., 2022). The simpler organic compounds are then used by bacteria for growth, contributing to the microbial CUE (Zhao et al., 2019). Thus, fungi and bacteria recruited by OAs could be more efficient in converting straw into biomass. This collaborative degradation strategy of complex macromolecular compounds into smaller compounds by fungi and bacteria validates the theory of the organic matter continuum proposed by Lehmann and Kleber (2015). This provides important insight into how OAs composed of specific organic compounds promote the growth and reproduction of specific microbial communities in agricultural ecosystems. OAs enhance the dominance of specific microbial populations, which allows them to degrade straw-C rapidly, and efficiently convert it into their biomass, improving SOC accumulation in agricultural soils.

## Conclusion

OAs significantly shifted the soil microbial community structure and increased the microbial use efficiency of straw-C. Compared with OAs with a low proportion of Hete_C, OAs with a high proportion of Hete_C significantly increased the microbial use efficiency of straw-C. F:B, *cbhI-GH48*, fungal diversity, and the relative abundance of *Ascomycota* were higher in OAs with a high proportion of Hete_C. Overall, our study suggests that adding OAs with a high proportion of Hete_C can increase the microbial use efficiency of straw-C by enhancing the abundance and diversity of fungi in the soil microbial community.

## Data availability statement

The datasets presented in this study can be found in online repositories. The names of the repository/repositories and accession number(s) can be found at: SRA data: PRJNA898503.

## Author contributions

JL, LC, and JZ conceived and designed the experiments. JL and LC performed the experiments and wrote the manuscript. JL analyzed the data. CZ, DM, GZ, and QN provided resources. All authors contributed to the article and approved the submitted version.

## Funding

This research was jointly supported by the Strategic Priority Research Program of Chinese Academy of Sciences (XDA28020203), the National Key Research and Development Program of China (2022YFD1500203), the National Natural Science Foundation of China (42177332), and the China Agriculture Research System (CARS-03-15 and CARS-52).

## Conflict of interest

The authors declare that the research was conducted in the absence of any commercial or financial relationships that could be construed as a potential conflict of interest.

The reviewer XH declared a shared parent affiliation with the authors to the handling editor at the time of review.

## Publisher’s note

All claims expressed in this article are solely those of the authors and do not necessarily represent those of their affiliated organizations, or those of the publisher, the editors and the reviewers. Any product that may be evaluated in this article, or claim that may be made by its manufacturer, is not guaranteed or endorsed by the publisher.

## Footnotes

^1^ https://www.ncbi.nlm.nih.gov/sra/PRJNA898503

^2^ http://rdp.cme.msu.edu/

## Supplementary material

SUPPLEMENTARY FIGURE S1 The richness indices of the bacterial **(A)** and fungal **(B)** communities in soils supplemented with different organic amendments (OAs) at days 7 and 28. Data are presented as means ± SD (n = 3). Error bars depict standard deviations. Lowercase letters indicate significant differences (P < 0.05) between the treatments. Ctrl (Control without OAs), LM (Addition of lignin-dominant OAs), MM (Addition of OAs with equal content of lignin and cellulose), and CM (Addition of cellulose-dominant OAs).

SUPPLEMENTARY FIGURE S2 Relative abundance of the major bacterial **(A)** and fungal **(B)** taxonomic groups at the phylum level in soil supplemented with different organic amendments (OAs) at days 7 and 28. Ctrl (Control without OAs), LM (Addition of lignin-dominant OAs), MM (Addition of OAs with equal content of lignin and cellulose), and CM (Addition of cellulose-dominant OAs).

SUPPLEMENTARY FIGURE S3 Effects of Alic_C, Alip_C, Arom_C, and Hete_C on fungal-to-ratios, cbhI-to-GH48 ratios, diversity and abundance of bacteria and fungi, dominant phylum, three main ecological clusters **(A)** and microbial use efficiency of straw-C **(B)**.

SUPPLEMENTARY FIGURE S4 Effects of bacterial and fungal diversity on microbial use efficiency of straw-C.
