# Supplementary material for: Organic amendments with high proportion of heterocyclic compounds promote soil microbiome shift and microbial use efficiency of straw-C
Source: Front Microbiol. 2023 Jan 18;14:1087709. doi: 10.3389/fmicb.2023.1087709 (PMC9889835; doi:10.3389/fmicb.2023.1087709)
Supplement: SUPPLEMENTARY FIGURE S1 — The richness indices of the bacterial (A) and fungal (B) communities in soils supplemented with different organic amendments (OAs) at days 7 and 28. Data are presented as means ± SD (n = 3). Error bars depict standard deviations. Lowercase letters indicate significant differences (P < 0.05) between the treatments. Ctrl (Control without OAs), LM (Addition of lignin-dominant OAs), MM (Addition of OAs with equal content of lignin and cellulose), and CM (Addition of cellulose-dominant OAs). [file Table_3.DOCX]

Supplementary Material

**Organic amendments and soil analysis**

The molecular structure of organic amendments (OAs) was measured using pyrolysis-GC/MS (MDGCMSMS TQ8050, SHIMADZU, Japan). Briefly, 0.5 mg of OAs was placed into a sample cup and heated at 600℃ for 5s in a pyrolysis furnace. The pyrolytic interface was set at 250℃, and the chromatographic temperature was increased from 40 to 320℃ (6℃ min^-1^). The initial 40 omatographic temperat1 min, then increased by 2℃ min^-1^ until 50℃, by 3℃ min^-1^ until 280℃, then by 10℃ min^-1^ until 300℃, and finally by 300℃ min^-1^ until 600℃. Helium gas flow was set at 1.0 mL min^-1^ in split mode during the pyrolysis progress. Pyrolysis substances with different molecular weights were separated using a fused-silica capillary J&W CP-Sil 5CB column (30 m×0.25 mm×0.25 μm) (Agilent Technologies Spain S.L. Las Rozas, Madrid). The mass spectrometer was conducted at 70 eV ionization energy and a mass detection range (m/z) of 45-800.

**Table S1** The organic compounds of organic amendments (OAs) were classified into four types based on the chemical structure type of organic carbon skeletons

| The type of organic compounds | Biogenic groups | Chemical structure type of organic carbon skeletons | Typical example |
| --- | --- | --- | --- |
| Alicyclic compounds | Cyclic hydrocarbon | Cyclic hydrocarbon chain | 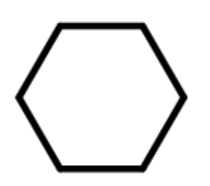 |
| Aliphatic compounds | Alkane, alkene, fatty acids | Lipid chain | 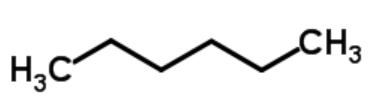 |
| Aromatic compounds | Lignin | Aromatic chain | 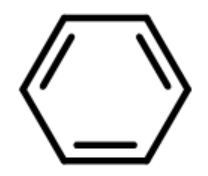 |
| Heterocyclic compounds | N-containing compounds; polysaccharid | Heterocyclic chain, N or O embedded in carbon chain | 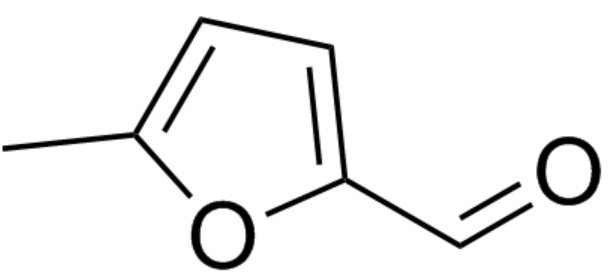 |

**Table S2** The ratios of gene abundance per unit time in soils with addition of organic amendments (OAs)

|  | **R1** | **R2** |
| --- | --- | --- |
| OAs with high proportion of Hete_C | **-2.96** | **0.52** |
| OAs with low proportion of Hete_C | **1.36** | **5.15** |

$R1=\frac{\Delta Fungi}{\Delta Bacteria}$ $R2=\frac{\Delta cbhI}{\Delta GH48}$

|Ri|<1: Faster change in denominator;

|Ri|>1: Faster change in numerator;

Negative value indicates inconsistent trends in numerator denominator changes


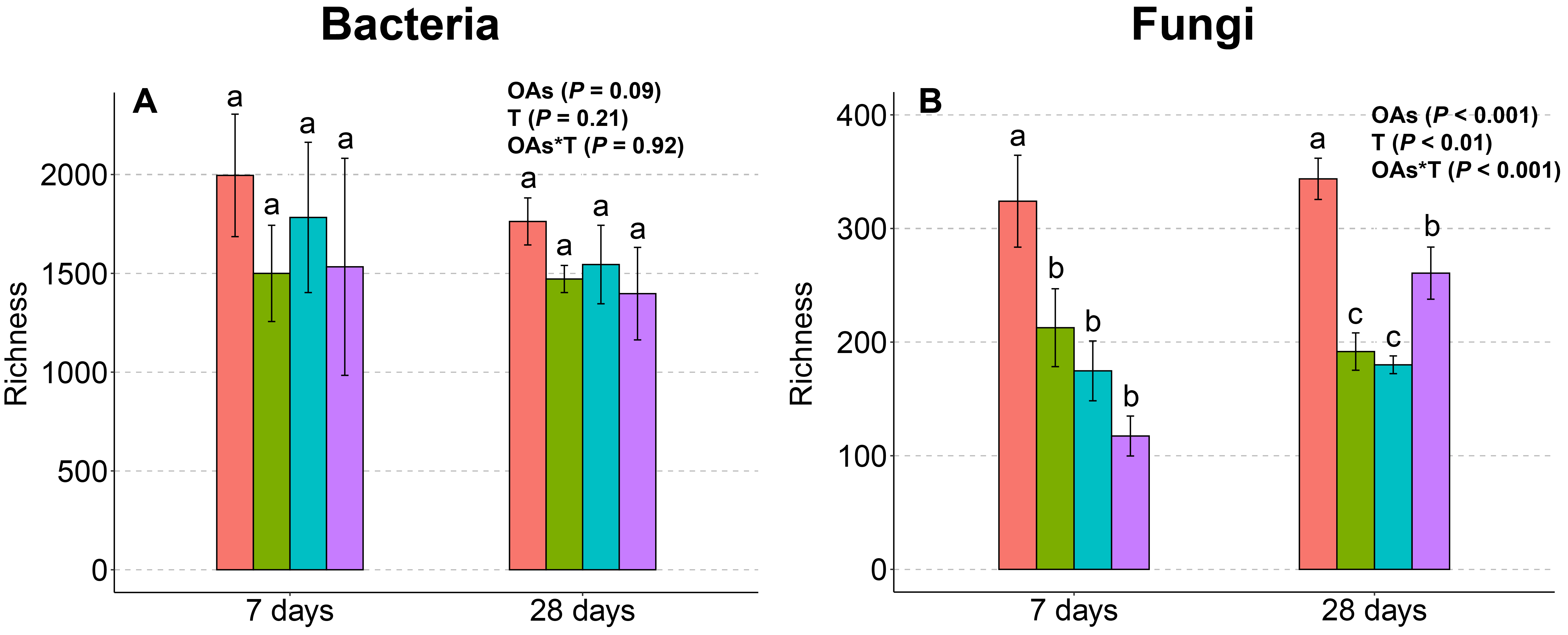


**Figure S1** The richness indices of the bacterial (A) and fungal (B) communities with different organic amendments (OAs) addition at 7 days and 28 days. Data are the means ± SD (n = 3). Error bars depict standard deviations. Lower-case letters indicate significant differences (*P* < 0.05) between the treatments.


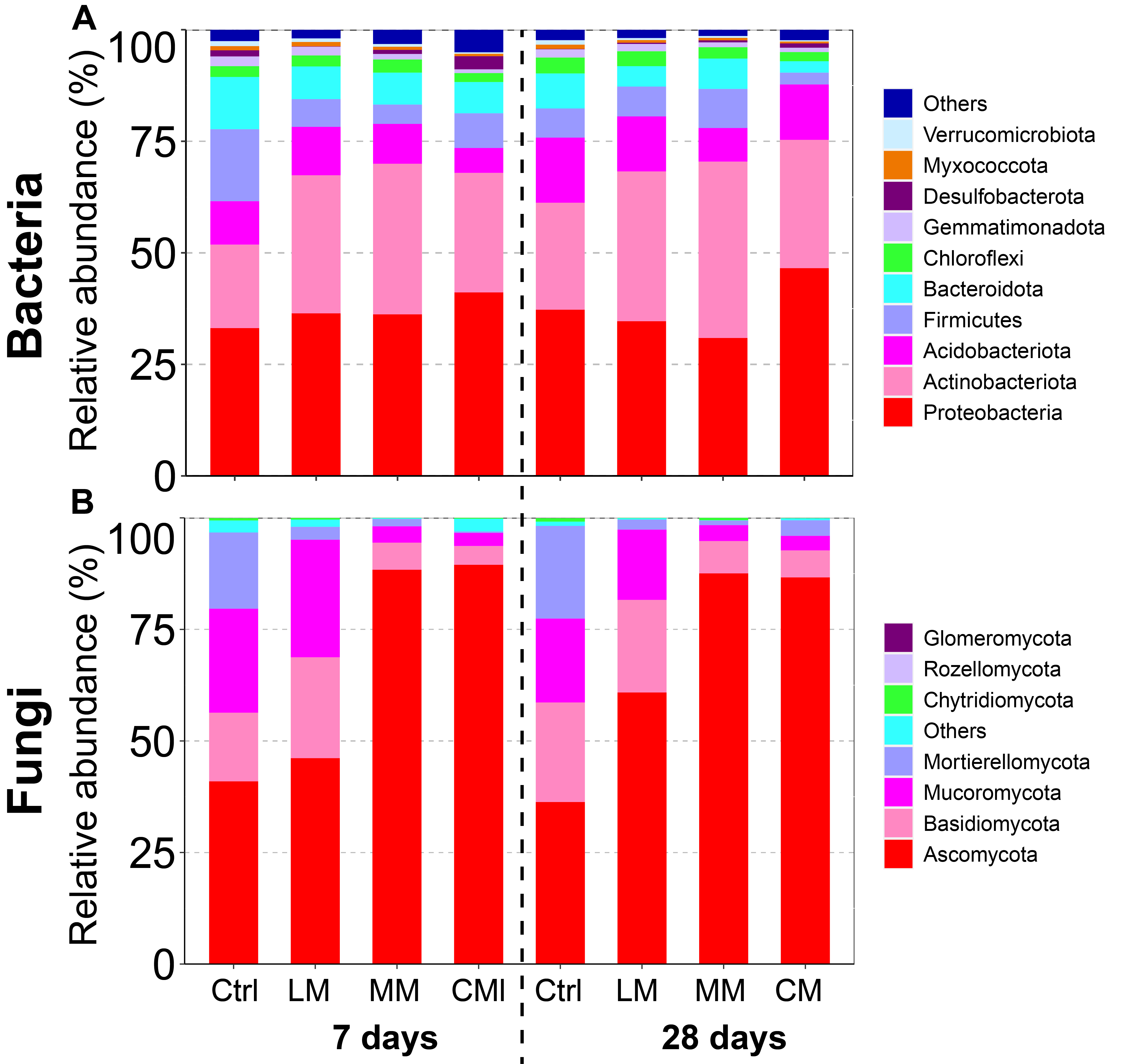


**Figure S2** Relative abundance of the major bacterial (A) and fungal (B) taxonomic groups at the phylum level with different organic amendments (OAs) addition at 7 days and 28 days.


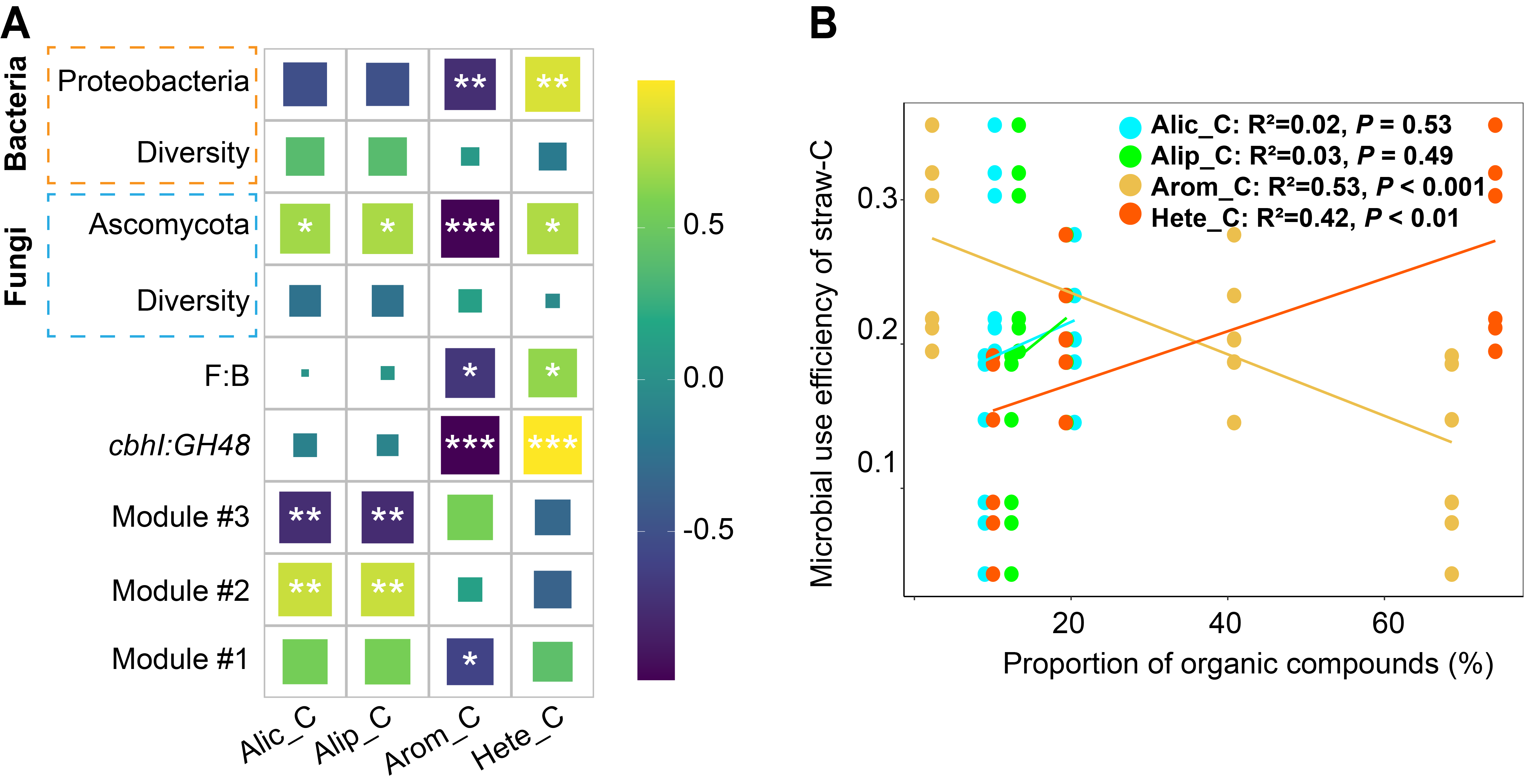


**Figure S3** Effects of Alic_C, Alip_C, Arom_C and Hete_C on fungal-to-ratios, cbhI-to-GH48 ratios, richness and diversity of bacteria and fungi, dominant phylum, three main ecological clusters (A) and microbial use efficiency of straw-C (B).


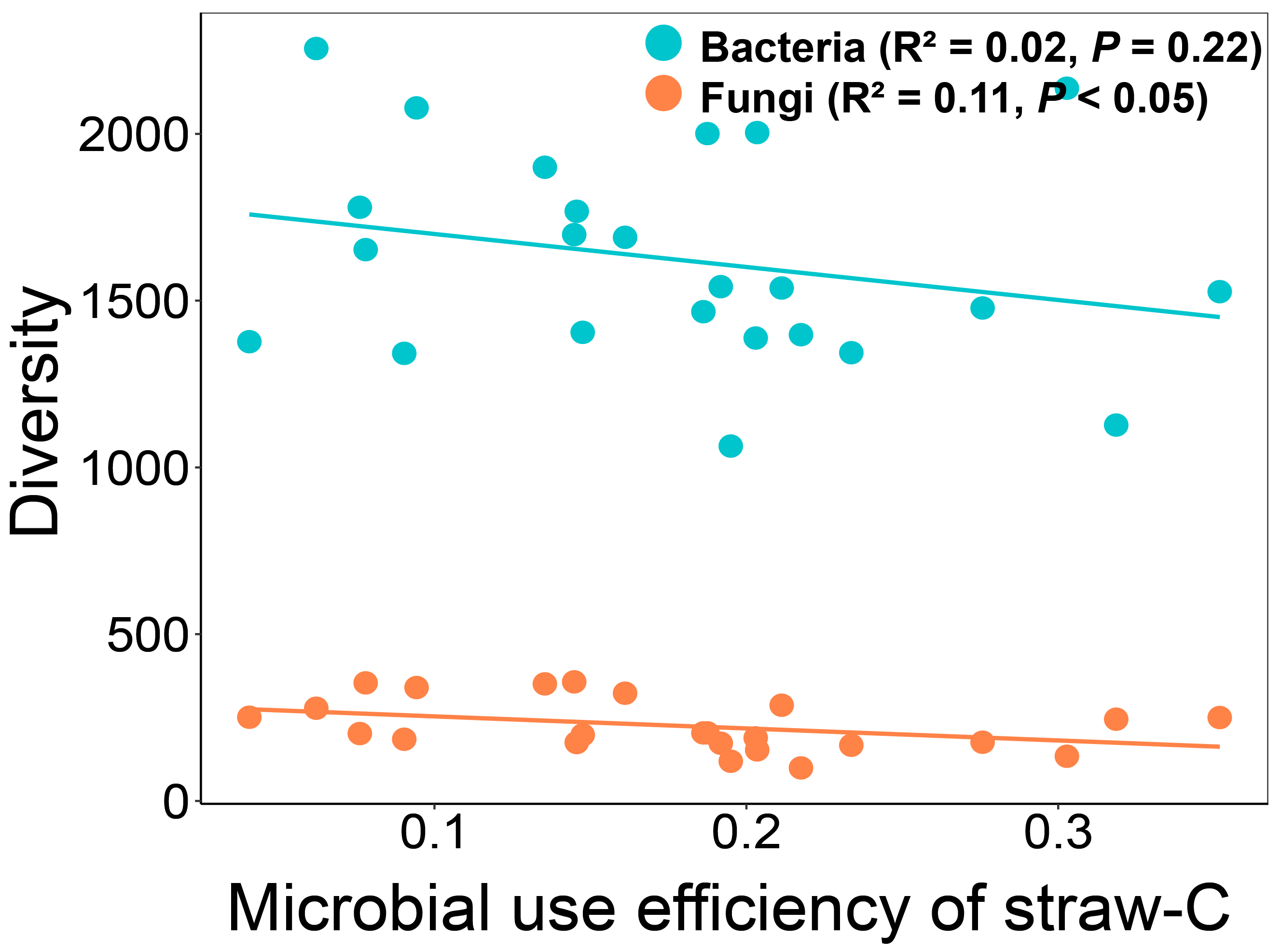


**Figure S4** Effects of diversity of bacteria and fungi on microbial use efficiency of straw-C.
